# Triatominae (Hemiptera, Reduviidae) in homes: Report of their occurrence in the municipality of Cruzeiro do Sul, Acre, South Western Amazon

**DOI:** 10.1590/0037-8682-0296-2020

**Published:** 2020-11-13

**Authors:** Madson Huilber da Silva Moraes, Adila Costa de Jesus, Fernanda Portela Madeira, Gilberto Gilmar Moresco, Jader de Oliveira, João Aristeu da Rosa, Luís Marcelo Aranha Camargo, Paulo Sérgio Bernarde, Dionatas Ulises de Oliveira Meneguetti

**Affiliations:** 1 Universidade Federal do Acre, Programa de Pós-Graduação em Ciências da Saúde na Amazônia Ocidental, Rio Branco, AC, Brasil.; 2 Universidade Federal do Acre, Centro Multidisciplinar, Cruzeiro do Sul, Campus Floresta, AC, Brasil.; 3 Ministério da Saúde, Departamento de Vigilância das Doenças Transmissíveis, Secretaria de Vigilância em Saúde, Brasília, DF, Brasil.; 4 Universidade Estadual Paulista Júlio de Mesquita Filho, Araraquara, SP, Brasil.; 5 Instituto Nacional de Epidemiologia da Amazônia Ocidental, Porto Velho, RO, Brasil.; 6 Centro de Pesquisa em Medicina Tropical de Rondônia, Porto Velho, RO, Brasil.; 7 Centro Universitário São Lucas, Departamento de Medicina, Porto Velho, RO, Brasil.; 8 Universidade de São Paulo, Instituto de Ciências Biomédicas, Monte Negro, RO, Brasil.; 9 Universidade Federal do Acre, Colégio de Aplicação, Rio Branco, AC, Brasil.

**Keywords:** Chagas disease, Epidemiology, Kissing bug, Vector

## Abstract

**INTRODUCTION:**

Triatomines are hematophagous insects that are important to public health since they are the vectors of American Trypanosomiasis. The objective of this study was to describe the occurrence of triatomines in homes in Cruzeiro do Sul, Acre, Brazil.

**METHODS:**

The specimens were collected by an active search inside homes and also by a passive search by the residents.

**RESULTS:**

A total of 55 triatomines were captured comprising of 5 species each of the genera *Rhodnius*, *Eratyrus*, and *Panstrongylus*. No colonies were detected, ruling out the possibility of domiciliation.

**CONCLUSIONS:**

Information on regional epidemiological dynamics contributes to the prevention and control of disease.

Triatomines, comprising of the family Reduviidae and the subfamily Triatominae, have epidemiological importance since they are vectors of *Trypanosoma cruzi*, an etiological agent of American Trypanosomiasis, also known as Chagas disease^1^, owing to their mandatory hematophagic habits^2^. The Triatominae subfamily currently represents 154 species (151 living species and three fossils) and is organized into 5 tribes and 18 genera^3^. 

In the state of Acre, Brazil, 11 species of triatomines are described belonging to four distinct genera: *Rhodnius robustus* Stål, 1872; *R. pictipes* Stål, 1872; *Panstrongylus geniculatus* Latreille, 1811; *Eratyrus mucronatus* Stål, 1859; *R. montenegrensis* Rosa et al., 2012; *R. stali* Lent, Jurberg & Galvão, 1993; *R. neglectus* Lent, 1954; *Triatoma sordida* Stål*,* 1859; *P. megistus* Burmeister, 1835; *P. lignarius* Walker, 1873; and *P. rufotuberculatus* Champion, 1899^4^.

Research carried out in this state has already described the occurrence of triatomines in homes^5^ ^,^ ^6^, however, no studies have investigated the occurrence of triatomines inside homes in the Juruá Valley region in the extreme south-western region of Brazil bordering Peru. Thus, the aim of this study was to describe the occurrence of triatomines and infection by trypanosomatids inside home environments in the municipality of Cruzeiro do Sul, Acre, Brazil.

The study area was the municipality of Cruzeiro do Sul (07º39'54"S 72º39'1"W) in the state of Acre, in the western Brazilian Amazon region. 

The collections were carried out from February 2016 to December 2018 (permanent license for zoo material collection, number 52260-1, from the Brazilian Institute of Environment and Renewable Natural Resources - IBAMA), both by passive and active searches. The passive search took place through the collection of triatomines by the residents who visualized supposed specimens inside their homes or in nearby areas and delivered them either to the Federal University of Acre (UFAC) or to Cruzeiro do Sul Endemic Management. The active search was carried out inside homes and nearby areas, in the same localities where triatomines were found through passive search and also in environments that provided a source of shelter or food for these insects, such as, stacks of bricks, wood, tiles, and animal breeding sites located near the dwellings.

The collected insects were sent to the Laboratory of Tropical Medicine (LABMEDT) of the UFAC for identification of the species through morphological characteristics using dichotomous keys described by Galvão^1^, Lent & Wygodzinsky^2^, and Rosa^7^. Triatomines that demonstrated similarities or aspects that made identification difficult were then sent to the Department of Biological Sciences, Faculty of Pharmaceutical Sciences, São Paulo State University “Júlio de Mesquita Filho” (UNESP), located in Araraquara, São Paulo, Brazil, for internal analysis of the genitals.

The analysis of trypanosomatids was performed in LABMEDT through an investigation of the intestinal content of the triatomines obtained by abdominal compression of previously diluted samples in a 0.9% physiological solution for fresh analysis and smear preparation, fixed with 0.1% triarylmethane, stained with 0.1% xanthene and 0.1% thiazine, and observed under 400× magnification with an optical microscope.

In the analysis period, 55 triatomines in 3 genera were captured (Figure 1). 

**FIGURE 1:**
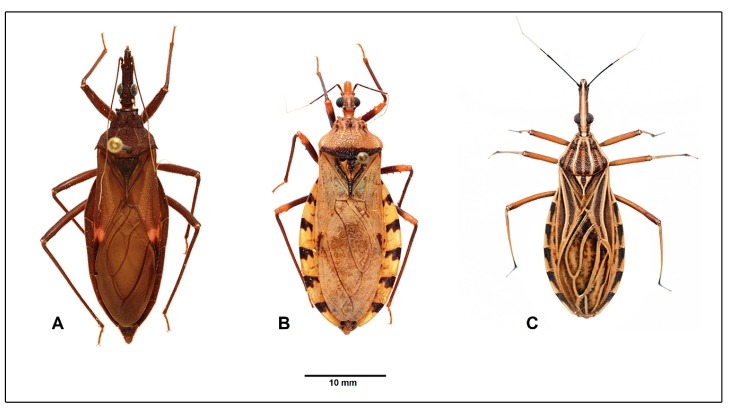
Species belonging to the genera of triatomines found in dwellings in the municipality of Cruzeiro do Sul, Acre. **(A)** *Eratyrus mucronatus*; **(B)** *Panstrongylus geniculatus*; **(C)** *Rhodnius montenegrensis*.

Table 1 shows the triatomine genera and various species collected during the study period, as well as, the frequency and positivity for trypanosomatids.

**TABLE 1:** Triatomines collected in dwellings, location, and positivity for trypanosomatids in the municipality of Cruzeiro do Sul, Acre, in the years of 2016, 2017, and 2018.

Year	Genus	Species	Peri/Intra^a^	N (%)	Infected (%)
2016	*Rhodnius*	*R. montenegrensis*	Peri	3 (5,5)	1 (33,3)
		*Rhodnius* sp.*	Peri/Intra	25(45,5)	1 (4,0)
2017	*Rhodnius*	*R. montenegrensis*	Intra	4 (7,3)	2 (50,0)
		*Rhodnius* sp.*	Peri/Intra	6 (10,9)	1 (16,7)
	*Eratyrus*	*E. mucronatus*	Peri	1 (1,8)	0
	*Panstrongylus*	*P. geniculatus*	Peri	1 (1,8)	0
2018	*Rhodnius*	*R. montenegrensis*	Peri/Intra	9 (16,4)	0
		*R. pictipes*	Intra	2 (3,6)	0
		*R. stali*	Intra	1 (1,8)	0
	*Eratyrus*	*E. mucronatus*	Intra	1 (1,8)	0
	*Panstrongylus*	*P. geniculatus*	Peri	2 (3,6)	0
Total				55 (100)	5 (9,1)

a **Peri/Intra:** Peridomicile/Intradomicile; * The species was not identified by the Heath´s Secretary staff due to a damage in the genitalia of the triatomine during the collection of feces during the analyses of infection by trypanosomatids.

With regards to the species captured in home environments, 33 species (60%) were captured around homes and 22 species (40%) were captured inside homes, of which more than half the species (73.3%) were collected in 2018. 

Twenty-three (41.8%) specimens were collected in urban areas and 32 (58.2%) in rural areas of the municipality. Of those captured in urban areas, the location which had the highest number of triatomines was the neighborhood of Aeroporto Velho, with 14 specimens, corresponding to 25.4% of the total insects collected in the study, followed by Miritizal and Tiro ao Alvo neighborhoods, with 5 (9.1%) and 4 (7.3%) triatomines, respectively (Figure 2). 

**FIGURE 2:**
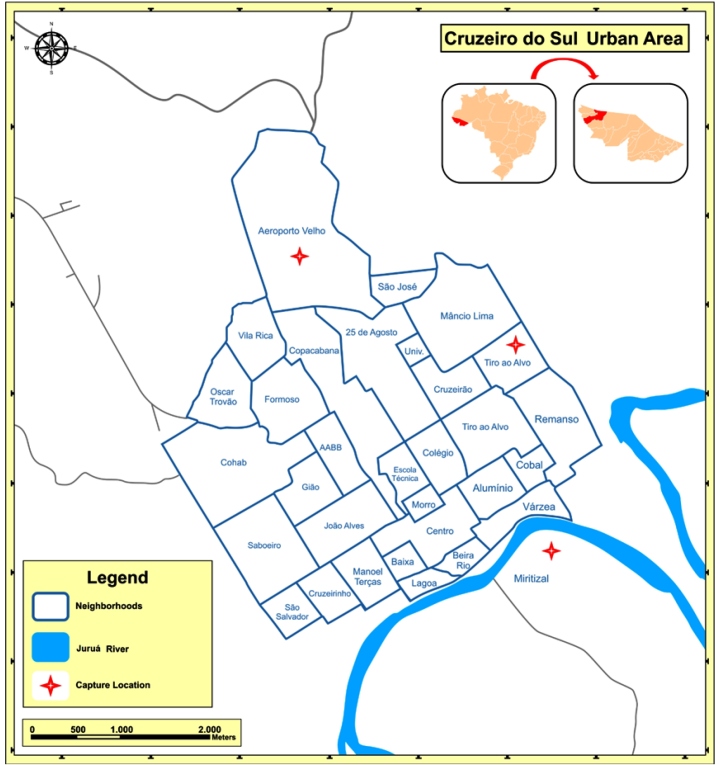
Urban neighborhoods of the municipality of Cruzeiro do Sul, Acre, with the capture locations of triatomines highlighted.

With regards to the triatomines collected in rural areas, the localities with the highest number of collected species were Boca do Môa, with 9 (16.4%) specimens, Vila Assis Brasil with 8 (14.5%), and Colônia Passo Fundo with 4 (7.3%). 

The genus *Rhodnius* predominated in this study and comprised 90.9% of the total collected insects. *R. montenegrensis* was the most captured species.

Triatomines belonging to the genus *Rhodnius* are usually associated with palm trees but can also be found in households as they are attracted by lights and are in search of food^8^, factors which might have influenced their capture rates in this study. The predominance of triatomines belonging to this genus was also observed in a survey conducted in wild and artificial environments both in rural and urban locations in the state of Manaus, in which more than 90% of all specimens captured were of the genus *Rhodnius* ^9^. *R. montenegrensis*, one of the most collected species in this study, has epidemiological relevance in the Amazon, mainly because its infection by *T. cruzi* ^10^ and *T. rangeli* ^11^ has already been described. 

The capture of specimens occurred mainly around homes, which corroborates a study conducted in rural communities in Ecuador where more than half of the collected triatomines were found around homes^12^.

With regards to the positivity indices for trypanosomatids, in a previous study carried out in the urban areas of Diamantina, a municipality located northeast of Minas Gerais, an infection rate of 19.6% was registered^13^. In the Amazon region of the state of Rondônia, 35.6% positivity for trypanosomatids was detected^14^. Both these previous studies registered higher rates than the observed values in this study.

There is an explanation for the occurrence of triatomines in the urban neighborhoods of the municipality studied. These regions are close to fragmented forest areas which resulted due to indiscriminate deforestation, where the presence of palm trees, which were already associated by the infestation of *T. cruzi-*infected triatomines^15^, might favor the entry of these vectors into houses. In rural areas, the predominance of specimens is related to the fact that the communities are in palm-rich forests; thus, the invasion of triatomes is presumed, increasing the possibility of contact between these insects and residents^10^. 

All the captured insects were in their adult stages and there was no detection of colonies, ruling out the possibility of domiciliation. However, the occurrence of vectors inside homes in urban areas is of concern, as this allows vector transmission of trypanosomatids. Although, it is important to highlight that in the Amazon region the main form of transmission is oral, mainly through juice and wine from palm fruits such as Açaí (*Euterpe oleracea*), Patuá (*Oenocarpus bataua*) and Bacaba (*Oenocarpus bacaba*). 

Therefore, it is suggested that health surveillance actions must be carried out, such as advising residents to implement measures to improve structural aspects of their homes to reduce the probability of vector entry. It is also important to consider the need to carry out new investigations, given the relevance of knowledge gained about regional epidemiological dynamics which can be used to plan public policies to prevent and control Chagas disease.
